# Prevalence of measles antibodies among health care workers in Catalonia (Spain) in the elimination era

**DOI:** 10.1186/1471-2334-13-391

**Published:** 2013-08-26

**Authors:** Luis Urbiztondo, Eva Borràs, Josep Costa, Sonia Broner, Magda Campins, José María Bayas, María Esteve, Angela Domínguez

**Affiliations:** 1 Public Health Agency, Generalitat of Catalonia, Roc Boronat, 81-95, 08005 Barcelona, Spain; 2 CIBER Epidemiología y Salud pública (CIBERESP), Madrid, Spain; 3 Hospital Clínic, University of Barcelona, Barcelona, Spain; 4 Hospital Vall d’ Hebrón, Autonomous University of Barcelona, Barcelona, Spain; 5 Hospital Germans Trías, Autonomous University of Barcelona, Badalona, Barcelona, Spain; 6 Department of Public Health, University of Barcelona, Barcelona, Spain

**Keywords:** Measles, Seroprevalence, Health care workers, MCV vaccination

## Abstract

**Background:**

Interruption of measles transmission was achieved in Catalonia (Spain) in 2000. Six years later, a measles outbreak occurred between August 2006 and June 2007 with 381 cases, 11 of whom were health care workers (HCW).

The objective was to estimate susceptibility to measles in HCW and related demographic and occupational characteristics.

**Methods:**

A measles seroprevalence study was carried out in 639 HCW from six public tertiary hospitals and five primary healthcare areas. Antibodies were tested using the Vircell Measles ELISA IgG Kit. Data were analyzed according to age, sex, type of HCW, type of centre and vaccination history.

The odds ratios (OR) and their 95% CI were calculated to determine the variables associated with antibody prevalence. OR were adjusted using logistic regression.

Positive predictive values (PPV) and the 95% confidence intervals (CI) of having two documented doses of a measles containing vaccine (MCV) for the presence of measles antibodies and of reporting a history of measles infection were calculated.

**Results:**

The prevalence of measles antibodies in HCW was 98% (95% CI 96.6-98.9), and was lower in HCW born in 1981 or later, after the introduction of systematic paediatric vaccination (94.4%; 95% CI 86.4-98.5) and higher in HCW born between 1965 and 1980 (99.0%; 95% CI 97.0-99.8). Significant differences were found for HCW born in 1965–1980 with respect to those born in 1981 and after (adjusted OR of 5.67; 95% CI: 1.24-25.91).

A total of 187 HCW reported being vaccinated: the proportion of vaccinated HCW decreased with age. Of HCW who reported being vaccinated, vaccination was confirmed by the vaccination card in 49%. Vaccination with 2 doses was documented in only 50 HCW, of whom 48 had measles antibodies. 311 HCW reported a history of measles.

The PPV of having received two documented doses of MCV was 96% (95% CI 86.3-99.5) and the PPV of reporting a history of measles was 98.7% (95% CI 96.7-99.6).

**Conclusions:**

Screening to detect HCW who lack presumptive evidence of immunity and vaccination with two doses of vaccine should be reinforced, especially in young workers, to minimize the risk of contracting measles and infecting the susceptible patients they care for.

## Background

Health care workers (HCW) are at greater risk of acquiring measles than the general public [1,2]. Transmission occurs from infected patients to staff and from infected staff to patients and colleagues. In both inpatient and outpatient settings, susceptible patients suffering other conditions, especially the elderly and severely ill patients in intensive care units, are at high risk of severe disease or death if infected with measles by a HCW [3,4]. The most effective preventive measure against measles is vaccination with two doses of measles-containing vaccine (MCV).

In Catalonia, a region in the northeast of Spain with 7.5 million inhabitants, MCV vaccination at 12 months was included in the immunization schedule in 1981. In 1988, the recommended age of MCV1 vaccination was raised to 15 months to improve effectiveness and MCV2 vaccination at 11 years of age was introduced to replace the monovalent rubella dose, in accordance with recommended measles elimination strategies. To reduce the number of cohorts vaccinated with a single dose, in 1998 MCV2 was advanced to 4 years [5]. Therefore, subjects born after 1981 should have received two doses of MCV, one at 12 or 15 months and another later in life.

The incidence of measles in Catalonia declined from 470 per 100000 inhabitants in 1983 to 1.01/100000 in 1997 and 0.5/100000 inhabitants in 1999. Elimination of endemic measles transmission was achieved in 2000 [6]. During the period 2000–2005, the incidence of measles in Catalonia was very low (51 cases in 6 years), and outbreaks were related to imported cases and affected few people [7].

A measles outbreak occurred in Catalonia between August 2006 and June 2007 with 381 cases (Incidence rate = 6.6/100000), mainly in children aged < 15 months. Transmission occurred in health care settings in 20% of the cases in which the location was identified. Among affected adults, 11 were HCW, of whom only one had received one dose of MCV, while the rest were not vaccinated [8]. In 2008, after the outbreak, the recommended age of MCV1 vaccination was changed back to 12 months, because protection of infants due to passively-acquired maternal antibodies is less long-lasting in vaccinated mothers not exposed to wild measles virus [9].

In 2008 and 2009, the incidence of measles was very low, with only 14 cases in small outbreaks linked to imported cases. Between November 2010 and September 2011, there was another outbreak with transmission among the native population that affected 305 people, mostly unvaccinated adults aged > 25 years, including 11 HCW, of whom one had received two doses of MCV [10]. Currently there is no continuous transmission of measles in Catalonia.

The objective of this seroprevalence study was to determine the level of protection against measles in HCW in Catalonia and the factors associated with evidence of measles immunity, in order to implement appropriate strategies to detect susceptible HCW and minimize their risk of contracting measles and infecting the susceptible patients they care for.

## Methods

The study was carried out using a convenience sample. Occupational Risk Prevention (ORP) services from the 10 primary healthcare areas and from 9 of the leading tertiary hospitals in Catalonia were asked to recruit patients. We considered HCW all persons, paid and unpaid, working in healthcare settings who had the potential for exposure to patients and/or to infectious materials. HCW included physicians, nurses, other clinical workers (nursing assistants, therapists, technicians, emergency medical service personnel, pharmacists, laboratory personnel, students and trainees) and non-clinical workers (clerical, house-keeping, laundry, security, maintenance, administrative staff and billing). HCW attending voluntary periodic health examinations between June 2008 and December 2010 were informed of the study and were recruited after written informed consent was obtained. The study was approved by the Ethics Committee of the University of Barcelona. Blood samples were obtained and demographic and epidemiological variables were collected using a questionnaire (age, sex, type of HCW, type of centre, history of having had measles disease and vaccination history) and completed by ORP physicians and nurses. If available, the vaccination card was also reviewed.

To determine long-lasting immunity to measles, IgG antibodies were studied using the Vircell Measles ELISA IgG Kit (Vircell SL. Granada. Spain). According to the manufacturer, the sensitivity and specificity of the method are 99% and 92%, respectively.

The prevalence of antibodies and the 95% confidence intervals (CI) were calculated using the exact binomial method.

The relationship between the dependent variable, measles antibodies, and the independent variables, age, sex, professional category and type of centre, was assessed using odds ratios (OR) and 95% CI. Odds ratios were adjusted using multiple logistic regression with two additional strategies: full model (i.e. with all candidate variables) and a backward selection procedure. The inclusion and exclusion criteria used were; p < 0.05 for model entry and p > 0.10 for output, according to Wald statistics. Statistical significance was established assuming an alpha error of 0.05.

The positive predictive value (PPV) and their 95% CI were calculated using a binomial distribution. The PPV of documented previous MCV vaccination was calculated as the number of HCW with both positive antibodies and documented MCV vaccination (2 doses) divided by the total of HCW with documented MCV vaccination (2 doses). The PPV of a reported history of measles infection was calculated as the number of HCW with both positive antibodies and a reported history of measles divided by the total of HCW with a reported history of measles. The 95% CI of the PPV were calculated using a binomial distribution.

Data processing and analysis were carried out using the SPSS v19.0 for Windows and R 2.13.0 (R Development Core Team 2011) programs.

## Results

Five of the 10 primary healthcare ORP and 6 of the 9 hospital ORP invited to participate accepted. The participating centres were located in 5 of the 7 Catalan health regions, representing 87.6% of the population.

A total of 639 HCW participated in the study, of whom 149 (23.3%) were male and 490 (76.7%) female. The median age of HCW was 41 years (Range 21 – 66). The distribution according to professional category showed a predominance of nurses (249; 29.6%) and physicians (189; 39.0%) followed by other clinical workers (86; 13.5%) and non-clinical workers (115; 18.0%). According to type of centre, 341 workers (53.4%) came from hospitals and 298 (46, 6%) from primary healthcare centres. Very few workers refused to take part in the study (> 5% in participating ORP). No HCW participating in the study was affected by the measles outbreak that began at the end of 2010.

The overall prevalence of measles antibodies was 98.0%, (95% CI 96.6-98.9) and was lower in HCW born in 1981 and after (94.4; 95% CI 86.4-98.4) than in those born between 1965 and 1980 (99%; 95% CI 97.0-99.8).

In the multivariate analyses, significant differences were found only for HCW born in 1965–1980 with respect to those born in 1981 and after, with an adjusted OR of 5.67 (95% CI: 1.24-25.91) (Table 1).

**Table 1:** Results of the bivariate and multivariate analyses of measles antibodies in healthcare workers

**Variable**	**n**	**Prevalence**	**Crude OR**	**p**	**Adjusted OR**	**p**
		**(95% CI)**	**(95% CI)**		**(95% CI)**	
*Year of birth*						
1981 and after	72	94.4 (86.4 – 98.5)	Reference		Reference	
1965 – 1980	292	99.0 (97.0 – 99.8)	5.67 (1.24 – 25.91)	0.02	5.67 (1.24 – 25.91)	0.02
1955 – 1964	164	97.0 (93.0 – 99.0)	1.87 (0.49 – 7.18)	0.36	1.87 (0.49 – 7.18)	0.36
1954 and before	111	99.1 (95.1 – 100)	6.47 (0.71 – 59.11)	0.09	6.47 (0.71 – 59.11)	0.09
All	639	98.0 (96.5 – 98.9)	-		-	
*Sex*						
Male	149	98.0 (94.3 – 99.6)	1.01 (0.28 – 3.73)	0.98		
Female	490	98.0 (96.3 – 99.0)	Reference			
*Professional group*						
Physician	189	98.9 (96.2 – 99.9)	2.5 (0.41 – 15.22)	0.32		
Nurse	249	97.6 (94.8 – 99.1)	1.08 (0.27 – 4.42)	0.91		
Other clinical workers	86	97.7 (91.9 – 99.7)	1.12 (0.18 – 6.88)	0.90		
Non-clinical workers	115	97.4 (92.6 – 99.5)	Reference			
*Type of centre*						
Primary health	298	98.7 (96.6 – 99.6)	1.99 (0.61 – 6.54)	0.25		
Hospital	341	97.4 (95.1 – 98.8)	Reference			

A total of 187 HCW reported being vaccinated, and the proportion of vaccinated decreased with age: 81.9% in HCW born in or after 1981, 39.0% in those born between 1965–1980, 6.1% in those born between 1955–1964 and 3.6% in those born in 1954 or before. In HCW who reported being vaccinated, vaccination was confirmed with the vaccination card only in 49%. Vaccination with 2 doses was documented in only 50 HCW, of whom 48 had antibodies against measles. A history of measles was reported by 311 HCW (48.7% of the total) and increased with age: 12.5% in HCW born in or after 1981, 43.5% in those born between 1965–1980, 59.8% in those born between 1955–1964 and 69.4% in those born in or before.

The PPV of documented measles vaccination with 2 doses with respect to positive serology was 96.0 (95% CI 86.3-99.5). The lowest PPV was found in HCW born in or after 1981 (95.5; 95% CI 77.2-99.9). No significant differences were found between HCW born after 1981 and the other birth cohorts (Table 2). The PPV of a history of measles with respect to positive serology was 98.7 (95% CI 96.7-99.6) (Table 3).

**Table 2:** PPV of having 2 documented doses of MCV for presence of measles antibodies

	**Measles vaccination**	
**Variable**	**Antibodies +/n**	**PPV**
		**(95% CI)**
*Year of birth*		
1981 and after	21/22	95.5 (77.2–99.9)
1965 – 1980	25/26	96.2 (80.4–99.9)
1964 – 1955	1/1	100 (2.5–100)
1954 and before	1/1	100 (2.5–100)
All	48/50	96 (86.3–99.5)
*Sex*		
Male	13/14	92.9 (66.1–99.8)
Female	35/36	97.2 (85.5–99.9)
*Professional category*		
Physician	22/22	100 (84.6–100)
Nurse	16/18	88.9 (65.3–98.6)
Other clinical workers	8/8	100 (63.1–100)
Non-clinical workers	2/2	100 (15.8–100)
*Type of centre*		
Primary health	10/11	90.9 (58.7–99.8)
Hospital	38/39	97.4 (86.5–99.9)

**Table 3:** PPV of a reported history of measles for the presence of measles antibodies

	**History of measles**	
**Variable**	**Antibodies +/n**	**PPV**
		**(95% CI)**
*Year of birth*		
1981 and after	9/9	100 (66.4–100)
1965 - 1980	127/127	100 (97.1–100)
1964 - 1955	95/98	96.9 (91.3–99.4)
1954 and before	76/77	98.7 (93.0–100)
All	307/311	98.7 (96.7–99.6)
*Sex*		
Male	60/60	100 (94.0–100)
Female	247/251	98.4 (96.0–99.6)
*Professional category*		
Physician	94/94	100 (96.2–100)
Nurse	120/123	97.6 (93.0–99.5)
Other clinical workers	34/35	97.1 (85.1–99.9)
Non-clinical workers	59/59	100 (93.9–100)
*Type of centre*		
Primary health	157/157	100 (97.7–100)
Hospital	150/154	97.4 (93.5–99.3)

## Discussion

The results of this study show that the prevalence of measles antibodies in HCW in Catalonia is higher than that found in other countries [11,12]. A study in a New York tertiary hospital [11] found a prevalence of 91%, and a Japanese study in which the majority of participants were tertiary hospital physicians found a prevalence of 92.6% [12]. The specific characteristics of hospitals do not seem to be the main explanation for the differences between these studies and ours (which also included primary healthcare workers) because we found no significant differences in the prevalence between hospital and primary healthcare workers. Likewise, we found no differences between professional categories.

In Catalonia, as in other countries, vaccination of all subjects without documented evidence of measles immunity is recommended. Sufficient evidence is considered as birth after 1965 with a documented history of physician-diagnosed measles, serologic evidence of immunity or written confirmation of receipt of two doses of MCV [5,13]. Adults born before 1966 are considered immune to measles, because the lack of vaccination and greater circulation of the virus resulted in near-universal exposure and the development of natural immunity [14].

Susceptible individuals may be found in population cohorts born between 1965 and 1980, as some persons may have avoided measles infection due to the reduction in the incidence and because they were not vaccinated [15]. However, this was not confirmed by the results of our study, as HCW from this age group had the highest prevalence of measles antibodies. Our results showed that the most susceptible group was HCW born after 1980, who should have received two doses of MCV. These findings are similar to those of Botelho-Nevers et al. [16], and Seo et al., who suggested that, in younger subjects, vaccination coverage remained low [11].

Although the objective of measles elimination in the European Region by 2010 was established, 120 outbreaks were reported throughout the region during the period 2005–2008, of which 17 had more than 250 cases, with 25 deaths [17]. Currently, the goal of elimination has been renewed as 2015 [18]. Therefore, improvements in vaccination coverage targeting all pockets of susceptible individuals and the early identification of and response to outbreaks are critical to achieving this target date for elimination in Europe [4].

In some developed countries, due to the low incidence of measles in the last twenty years, exposure and the risk of infection of non-vaccinated subjects, including HCW, has been minimal. As vaccination coverages increase and the incidence of measles declines, nosocomial transmission is likely to become an increasingly important source of measles virus in the population [19-23]. In these circumstances, physicians are less familiar with diagnosing measles, and delays occur in the diagnosis and laboratory confirmation of measles [24-26], increasing the risk of nosocomial transmission. HCW have been affected by many outbreaks and their role in measles transmission is key on many occasions [15,27,28]. Given the potential severity of measles and the ease of transmission in healthcare centres, vaccination of susceptible HCW is essential to control nosocomial infection and achieve progress in the elimination strategy [4,21].

Because HCW are at extremely high risk of acquiring measles from patients or transmitting measles to patients and co-workers in medical settings, in addition to the universal vaccination of children, the vaccination of susceptible individuals working in healthcare facilities with two doses of vaccine separated by an interval of at least 28 days is currently recommended [5,12,29].

Most HCW are immune to measles, but many cannot provide sufficient accessible evidence of documented immunity. If outbreaks occur, these HCW should be temporarily taken off health care work, which may cause severe logistic and financial problems [24,26]. In circumstances in which HCW state they know their history [30,31], undocumented information is clearly not sufficient to justify overriding these problems. This is true even for situations in which the PPV for immunity of having had measles or being vaccinated are high, as in the present study.

In the era of measles elimination, the goal is 100% immunity in populations at high risk of acquiring measles, such as HCW. The risk of acquiring measles is estimated to be 13 to 19 times higher for susceptible HCW than for the general population [4]. The criteria accepted as sufficient evidence of immunity in the general population may be insufficient in HCW, especially in younger age groups. These criteria should be reviewed, replacing the required documentation of physician-diagnosed disease as a evidence of measles immunity by laboratory confirmation of measles [13,21,24,32]. Those HCW who cannot provide proof of laboratory-confirmed measles or receipt of 2 doses of MCV should receive a full course of vaccination [4].

In Catalonia, the priority should be the availability of information on HCW measles immunity. Measles serology should be required in all HCW born after 1966 without documented evidence of vaccination with two doses of MCV or laboratory confirmation of the disease. The data should be stored and easily accessible, in computerized occupational records [4,24]. The vaccination card demonstrated limited usefulness in confirming vaccination in our study, suggesting that the undocumented histories reported by HCW have little validity. Another preoccupation is the possible limitation of the vaccination history of two doses as a criterion to ensure immunity. Although this information was only obtained in 50 HCW, serologies were negative in 4%. In addition, in the 2010 outbreak in Catalonia, one of the 11 HCW affected was an emergency room physician who had 2 documented doses of MCV [10].

A lesser priority should be routine review of the situation of unvaccinated HCW born before 1966 who lack laboratory evidence of measles immunity or laboratory confirmation of measles, in whom the recommendation of two doses of MCV should be strongly considered.

One limitation of this study is that, as we used a convenience sample, the results may not be generalizable to all HCW in Catalonia. Likewise, the serological study was made in HCW who voluntarily attended ORP health examinations: therefore, the prevalence of measles immunity in the study subjects may differ from that of HCW who did not attend these health examinations. However, the large sample size, which included hospital and primary healthcare centre workers from 5 of the 7 Catalan health regions, added to the fact that less than 5% of HCW invited to participate refused, suggest that our results may reflect the true situation in many Catalan health centres.

## Conclusion

Although most HCW have immunity against measles, everybody working in medical facilities should have evidence of this immunity [33]. Optimal preparedness for measles exposure includes ensuring that all HCW have documented and easily retrievable measles immunity records to guide case management and outbreak response [24]. Vaccination of susceptible subjects with two doses of MCV should be reinforced, especially in young workers, to minimize the risk of contracting measles and infecting the susceptible patients they care for.

## Competing interests

All authors declare that they have no conflicts of interest.

## Authors’ contributions

All the authors participated in the design, implementation, analysis and interpretation of the results of the study. AD was the principal investigator and secured funding. LU and AD drafted the report. JC performed the laboratory analysis. EB, MC, JMB, and ME supervised the study and reviewed the draft article. SB conducted the statistical analysis. The other members of the Working Group contributed to recruitment of subjects, data collection and interpretation of the results. All authors read and approved the final manuscript.

## Pre-publication history

The pre-publication history for this paper can be accessed here:

http://www.biomedcentral.com/1471-2334/13/391/prepub
